# Effect of Fluoride Coatings on the Corrosion Behavior of Mg–Zn–Ca–Mn Alloys for Medical Application

**DOI:** 10.3390/ma16134508

**Published:** 2023-06-21

**Authors:** Tiberiu Bita, Aurora Antoniac, Ion Ciuca, Marian Miculescu, Cosmin Mihai Cotrut, Gheorghe Paltanea, Horatiu Dura, Iuliana Corneschi, Iulian Antoniac, Ioana Dana Carstoc, Alin Danut Bodog

**Affiliations:** 1 Faculty of Material Science and Engineering, University Politehnica of Bucharest, 313 Splaiul Independentei, District 6, 060042 Bucharest, Romania; tiberiu.bita@yahoo.com (T.B.); antoniac.aurora@gmail.com (A.A.); ion.ciuca11@gmail.com (I.C.); marian.miculescu@upb.ro (M.M.); cosmin.cotrut@upb.ro (C.M.C.); 2 Faculty of Electrical Engineering, University Politehnica of Bucharest, 313 Splaiul Independentei, District 6, 060042 Bucharest, Romania; gheorghe.paltanea@upb.ro; 3 Faculty of Medicine, Lucian Blaga University of Sibiu, 2A Lucian Blaga Street, 550169 Sibiu, Romania; ioana.carstoc12@gmail.com; 4 Romfire Protect Solution S.R.L., 39 Drumul Taberei, District 6, 061359 Bucharest, Romania; iulicorneschi07@gmail.com; 5 Academy of Romania Scientist, 54 Splaiul Independentei, 050094 Bucharest, Romania; 6 Faculty of Medicine and Pharmacy, University of Oradea, 10 P-ta 1 December Street, 410073 Oradea, Romania; alinbodog@gmail.com

**Keywords:** Mg–Zn–Ca–Mn magnesium alloy, corrosion, fluoride conversion, surface

## Abstract

The most critical shortcoming of magnesium alloys from the point of view of medical devices is the high corrosion rate, which is not well-correlated with clinical needs. It is well- known that rapid degradation occurs when an implant made of Mg-based alloys is placed inside the human body. Consequently, the implant loses its mechanical properties and failure can occur even if it is not completely degraded. The corrosion products that appear after Mg-based alloy degradation, such as H_2_ and OH^−^ can have an essential role in decreasing biocompatibility due to the H_2_ accumulation process in the tissues near the implant. In order to control the degradation process of the Mg-based alloys, different coatings could be applied. The aim of the current paper is to evaluate the effect of fluoride coatings on the corrosion behavior of magnesium alloys from the system Mg–Zn–Ca–Mn potentially used for orthopedic trauma implants. The main functional properties required for the magnesium alloys to be used as implant materials, such as surface properties and corrosion behavior, were studied before and after surface modifications by fluoride conversion, with and without preliminary sandblasting, of two magnesium alloys from the system Mg–Zn–Ca–Mn. The experimental results showed that chemical conversion treatment with hydrofluoric acid is useful as a method of increasing corrosion resistance for the experimental magnesium alloys from the Mg–Zn–Ca–Mn system. Also, high surface free energy values obtained for the alloys treated with hydrofluoric acid correlated with wettability lead to the conclusion that there is an increased chance for biological factor adsorption and cell proliferation. Chemical conversion treatment with hydrofluoric acid is useful as a method of increasing corrosion resistance for the experimental Mg–Zn–Ca–Mn alloys.

## 1. Introduction

Magnesium-based alloys are more and more used as temporary orthopedic implants. They are characterized by high biocompatibility, biodegradability, and adequate physical and mechanical properties such as Young’s modulus of 45 GPa, a value that is close to that of 20 GPa related to the human bone, and a density between 1.74–1.84 g/cm^3^, which can be considered almost equal to that of human bone [1,2,3]. When implants made from Mg-based alloys are used to fix small or large hard tissue defects, the stress-shielding effect, which is present in the case of permanent medical devices made of inert metals such as stainless steel, Co-Cr, Co-Cr-Mb, or titanium (Ti) and its alloys [4], does not appear. In these cases, good bone regeneration due to proper osteoblast adhesion and proliferation can be noticed.

The advantages of Mg-based alloys are numerous. The first one is related to Mg’s low density, good rigidity, and high strength. It was proven that Mg is one-third lighter than Al, and its stiffness in the cast state is equal to that of cast Al, so as a consequence, the mechanical strength exhibits a higher value. Adequate shock absorption and the good toughness of the material were also evidenced [1]. Magnesium has good thermal conductivity, dimensional stability, and castability, and it can be recycled and reused in new implant fabrication. Other important characteristics that can be mentioned are the excellent machinability of the material and its good damping performance [1,2].

The alloying elements and the corresponding microstructural features influence the properties of Mg-based alloys. A classification of Mg-based alloys is made as a function of alloying elements such as zinc (Zn), calcium (Ca), zirconium (Zr), silver (Ag), rare earth (RE), and aluminum (Al). The result consists of seven binary alloys as follows: Mg–Zn, Mg–Ca, Mg–Zr, Mg–Ag, Mg–RE, Mg–Y, and Mg–Al [5].

The main shortcoming of the Mg-based alloys is their flammability because Mg exhibits a high affinity for oxygen. It is challenging to use Mg powder in powder metallurgy or additive manufacturing methods because it has high surface energy and an inert work atmosphere is needed. Another disadvantage is Mg’s poor plasticity at room temperature, which is an effect of the material’s closely packed hexagonal structure characterized only by one slip plane and three slip coordinate systems at 23 °C. In the case of temperatures higher than 250 °C, a supplementary slip surface occurs, so the plastic deformation becomes predominant [6]. The most critical shortcoming of Mg from the point of view of medical devices is its high corrosion rate. It is well known that rapid degradation occurs when an Mg-based implant is placed inside the human body, and loss of mechanical properties and implant failure can occur. It can be concluded that the corrosion rate must be carefully checked in order to avoid such cases [7]. The Mg corrosion products, such as H_2_ and OH^−^, can have an essential role in decreasing biocompatibility due to the H_2_ accumulation process in the implant neighborhood. As a result, the gas pocket formation negatively affects human tissues [8,9]. Supplementary, the OH^−^ ions can be directly linked to an alkalinization of the surface, which plays an important role in the cells’ damage [10,11,12,13].

Today, efforts are made to correct the three above-mentioned disadvantages of Mg: low mechanical strength, high pyrophoric character, and increased corrosion rate. The last one is the most important in the medical implant field, and the Mg-based alloy degradation phenomenon in different physiological media should be fully understood to be correctly addressed. It is mandatory to use physiological conditions when materials are immersed in simulated body fluids [14]. By introducing cell culture conditions, accelerated degradation of the materials could be observed by monitoring the osmolality [15]. The best simulated physiological solutions used in research practice proved to be Dulbecco’s Modified Eagle’s Medium (DMEM), Simulated Body Fluid (SBF), Hank’s balanced salt solution (HBSS), Earle’s balanced salt solution (EBSS), Kirkland’s biocorrosion medium (KBM), Eagle’s Minimum Essential Medium (E-MEM), and Minimum Essential Medium (MEM) [15]. The NaCl solution is another important testing medium because it facilitates the degradation of Mg-based alloys, so mechanical properties and hydrogen emissions can be investigated based on an accelerated model. Yang et al. [16] analyzed the micro galvanic corrosion of Mg–Ca and Mg–Al–Ca alloys in NaCl and Na_2_SO_4_ solutions. It was reported that rapid corrosion of the secondary phases existent in the above-mentioned alloys occurred in the case of NaCl medium. Another study [17] presented a localized corrosion process related to NaCl solutions. The authors stated that the incubation and growth of active zones placed in the film-free region are influenced by Cl ion concentration. The NaCl solution is adequate for Mg-based alloy degradation investigations because, inside the human body, there is a high quantity of chloride ions due to human nutrition or air because salt is used for road management and is present in marine environments. Hornig et al. [18] observed in the case of Mg–Y–Zn alloy that for low NaCl concentration solutions, the corrosion effects are localized since at higher concentrations pitting appears and, supplementary to the pits, the micro galvanic corrosions conduct a filiform corrosion attack. It was concluded that, based on NaCl concentration, the corrosion process can vary from pitting to filiform corrosion [18]. The type of solution is important, and it must be chosen in accordance with the medical application in which the Mg-based implant will be used because different degradation rates of the alloy can be obtained [19]. The detailed chemical composition of the most involved fluids in research for Mg-based corrosion analyses is described by J. Gonzales et al. [15]. Almost all the physiological media contain inorganic ions such as sodium (Na^+^), potassium (K^+^), chloride (Cl^−^), magnesium (Mg^2+^), calcium (Ca^2+^), and chemical radicals such as HCO^3−^, HPO_4_^2−^, H_2_PO_4_^−^, and SO_4_^2−^ [15,20,21,22,23,24,25,26,27,28,29].

In order to control the degradation process of the Mg-based alloys, surface modifications are applied [30,31]. Some of the most commonly used methods are alkaline heat treatment, self-passivation, and hydrothermal treatment [32]. The self-passivation consists of a thin oxide film formation on the alloy surface, but some studies show that the magnesium oxide (MgO) does not exhibit sufficient protective quality, and alloying of magnesium with different metals is needed [33]. Based on hydrothermal treatment made by soaking the alloy in deionized water or NaOH, a uniform layer of Mg(OH)_2_ with hydrophilic character beneficial to cell adhesion and proliferation appears [34]. A Ca-P apatite layer on the Mg surface is obtained through alkaline heat treatment due to the effect of different solutions, such as SBF or NaHCO_3_. Important surface modifications are also chemical ones. Chemical passivation represents the easiest method to change the Mg-based alloy surface properties. Another technology called reaction with ionic liquids consists of the physisorption of liquid anions on the Mg surface and the formation of a thin layer of nanometer order [35]. Chemical conversion coating based on cerium- [36], titanate-, phosphate- [37], and fluoride-conversion coating on Mg exhibit many advantages such as corrosion rate reduction, control over pH increase and hydrogen gas accumulation, high biocompatibility of the Mg-based alloy surface, promotion of Ca-P formation in the case of the last conversion treatment, and good cellular response [2]. Three ways to prepare a fluoride conversion coating were identified in the literature. The first consists of Mg-based alloy dipping in hydrofluoric acid (HF), the second is based on vacuum evaporation deposition, and the last is characterized by sample immersion into Na[BF_4_] molten salt [1,2]. Except for the third technology in the first two cases, a highly biocompatible MgF_2_ is formed. For the last treatment, it was noticed that the supplementary apparition of a toxic layer of NaMgF_3_ must be removed by boiling the coated Mg alloy in distilled water [38,39].

Bioactive glass coatings and biodegradable polymer coatings are frequently used in tissue engineering because of their high bioactivity, good osteoconductivity, and controllable biodegradability [40]. In the electrochemical surface modification class, the most commonly used techniques are anodizing [41] and micro-arc oxidation. Other methods are cathodic plasma electrolysis [42], physical vapour deposition [43], ion implantation [44], and sputtering [45].

Mechanical surface treatment is considered a proper approach to modifying and controlling the material’s bioactivity and degradation. The surface mechanical attrition [46] consists of a severe plastic deformation that induces compressive residual stresses into the Mg-based alloy surface. Another mechanical method is friction stir processing, which can improve the material’s ductility without affecting its mechanical properties. Based on this technology, welding defects such as cracks, porosity, and evaporative loss can be removed [47,48]. Abrasive water jet machining is considered an innovative method that can modify the surface roughness of the alloy and improve corrosion resistance. The shot peening is similar to surface mechanical attrition, which introduces compressive residual stress based on smaller balls projected into a substrate with high velocity [49]. One of the most facile mechanical surface modifications is sandblasting with hard particles such as Al_2_O_3_. To control the surface roughness, the particles’ kinetic energy, size, and shape are very important. During the sandblasting process, the particles shoot, generating supplementary kinetic energy proportional to the particle velocity, volume, density, and square. Sandblasting is a technology that increases surface roughness and facilitates the osteointegration of the implant. In Table 1, there are some examples of different surface treatments that reduce the Mg-based alloy corrosion rate and improve their mechanical properties and biocompatibility.

The aim of the current paper is to evaluate the effect of fluoride coatings on the corrosion behavior of Mg–Zn–Ca–Mn alloys for medical applications. The main functional properties required for the magnesium alloys to be used as implant materials, like surface properties and corrosion behavior, were determined before and after surface modifications by fluoride conversion (and sandblasting) of two Mg–Zn–Ca–Mn alloys. We consider that fluoride coatings on the Mg–Zn–Ca–Mn alloys could be a solution to modulate the corrosion behavior and adapt the biodegradation process of the magnesium-based alloys to the clinical needs in orthopedic surgery.

## 2. Materials and Methods

In this study, samples of dimensions 15 mm × 15 mm × 5 mm were cut from two Mg–Zn–Ca–Mn alloys (fabricated by stir casting using as raw materials Mg (high purity, 99.99%), Zn (high purity, 99.99%), Ca (high purity, 99.99%) and Mn (high purity, 99.99%)-Merck, Darmstadt, Germany) with the composition presented in Table 2.

The samples were polished up to 1200 grit with silicon carbide abrasive papers (SiC), then rinsed in acetone, ethanol, and distilled water successively [2].

Two types of surface modification were applied to the investigated ZMX100 and ZMX410 alloys, a chemical conversion using hydrofluoric acid (HF), and a sandblasting process (Mini Sandblaster, Caloris, Bucharest, Romania). For the chemical conversion treatment, the samples of each type of alloy were immersed in hydrofluoric acid of 40 wt% (Sigma-Aldrich, Darmstadt, Germany), at room temperature, for 24 h. After the treatment, the samples were rinsed with deionized water and dried. Alumina (Al_2_O_3_, purchased from Poka, Bucharest, Romania) was used for sandblasting the Mg–Zn–Ca–Mn alloy samples; the process was carried out from a distance of 10 mm with a pressure of 0.3 MPa, for 20 s. After the sandblasting, the treated surface was blown with dry air at a pressure of 3 atm. and then ultrasonicated in isopropyl alcohol for 20 min (Ultrasonic cleaner device Sonorex Super RK 106, Bandelin electronic GmbH & Co. KG, Berlin, Germany). The coding of the obtained experimental samples is presented in Table 3.

### 2.1. Microstructural and Surface Characterization

The microstructure of the investigated ZMX100 and ZMX410 alloys was observed using an Olympus BX51 optical microscope (Olympus Life and Materials Science Europa GMBH, Hamburg, Germany) after the surface had previously been etched with a solution consisting of 5 mL of acetic acid, 6 g of picric acid, 10 mL of distilled water, and 100 mL of ethanol. The surface morphology of the experimental samples was evaluated with a Philips XL 30 ESEM TMP scanning electron microscope (FEI/Phillips, Hillsboro, OR, USA) with energy dispersive spectroscopy (EDS). A Panalytical X-Pert PRO Diffractometer (Malvern Panalytical, Malvern, UK) was used to identify the phases in the magnesium alloy samples’ structure.

The material’s wettability was investigated based on contact angle measurements. The device used for sample characterization was the Krüss Drop Shape Analyzer—DSA100 (A. Krüss Optronic GmbH, Hamburg, Germany), which permits experiments with three wetting agents as follows: water, diiodomethane (DIM), and ethylene glycol (EG). The measurements were performed at room temperature of 23 ± 5 °C and humidity of 45 ± 5%. We had 12 samples of each magnesium-based alloy (3 samples for each surface treatment) and made an average of 3 determinations per sample or wetting agent. All the obtained images were manually analyzed with the ImageJ 1.50 software (National Institutes of Health, Bethesda, MD, USA). To compute the surface free energy (SFE), we applied the Owens, Wendt, Rabel, and Kaelbe (OWKR) methods [50].

### 2.2. Corrosion Behavior by Electrochemical and Immersion Test

Electrochemical tests and polarization resistance (Tafel plots) were performed with a PARSTAT 4000 Potentiostat/Galvanostat equipment (Princeton Applied Research, Oak Ridge, TN, USA), at 37 ± 0.5 °C, in NaCl solution 0.9 wt% (Sodium chloride purchased from Sigma-Aldrich, Darmstadt, Germany). The exposed area of all experimental samples was 1 cm^2^. A typical three-electrode cell was used with a platinum electrode used as the counter electrode (CE), the sample as the working electrode (WE), and a saturated calomel (SCE) as the reference electrode (RE). Before polarization resistance experiments, the open circuit potential was monitored for 1 h. All measurements were conducted according to the ASTM G5-14e1 standard.

The immersion test of the experimental samples was carried out in 50 cm^3^ of sodium chloride with a pH value of 7.0 at 37 ± 0.5 °C. The test evaluates the corrosion behavior through weight loss determination after 1, 3, 5, 7, and 14 days of immersion. During the test, the NaCl solution was changed every day at the same hour. Weight loss was calculated based on the following equations:

(1) $$WL\;\left(\%\right)=\frac{W_{i}-W_{f}}{W_{i}}\times 100,$$

where: *W_i_* is the initial mass value recorded at the beginning of the experiment; *W_f_* is the final mass value at the end of the experiment.

## 3. Results and Discussion

### 3.1. Microstructural and Surface Analysis

**Figure 1:**
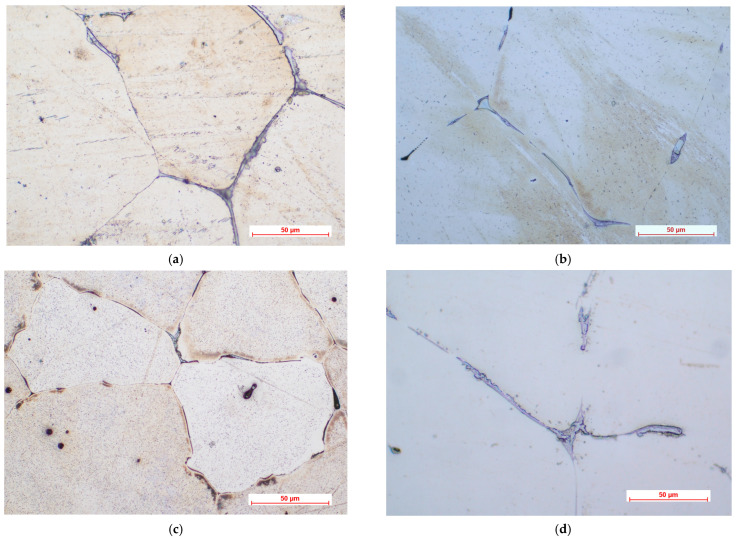
Optical microscopic images of ZMX100 alloy (**a**,**b**) and ZMX410 alloy (**c**,**d**).

Figure 1 shows the optical micrographs corresponding to ZMX100 and ZMX410 alloys at different magnifications. The structure is made up of large and relatively uneven α-Mg polyhedral grains, in which there are precipitated secondary phases, as well as a separated phase at the grain boundary, a eutectic.

As is known, Ca, Mn, Zr, Y, and Sr are the most used elements added to the Mg–Zn-based alloys to determine the microstructure refinement that improved the mechanical properties of the newly obtained alloys [51,52,53,54,55,56,57]. Since Ca and Mn are found in approximately equal proportions in the ZMX100 and ZMX410 alloy compositions, no significant differences were evident in the alloy’s microstructure in terms of the size of the obtained grains. Increasing the Zn content from 1.3% (in ZMX100 alloy) to 4.3% by weight (in ZMX410 alloy), a slight decrease in grain size is observed (Figure 1a,c). This aspect indicates that Zn can refine the microstructure of the ZMX410 alloy, as was also highlighted by H.R. Bakhsheshi-Rad et al. [58]. Also, the higher Zn content induces a better outline of the eutectic, highlighted as an almost continuous network at the limit of the grain boundary.

The phase diagram of the Mg–Zn–Ca ternary alloy with 2 wt% Zn highlighted the presence of α-Mg, Mg_2_Ca, and Mg_6_Zn_3_Ca_2_ phases. By increasing the Zn content to 4 wt%, only α-Mg and Mg_6_Zn_3_Ca_2_ phases are formed. Through Mn addition, the microstructure of the quaternary alloys is similar to that of the ternary alloys without Mn; the only difference noticed was the presence of α-Mn phase precipitates [59,60,61].

**Figure 2:**
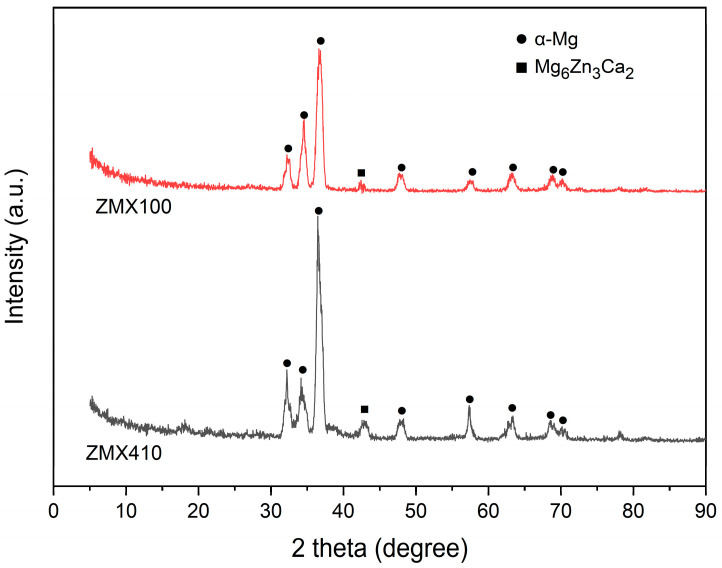
X-ray diffraction patterns of the as-cast ZMX100 and ZMX410 alloys.

From the XRD results (Figure 2), it can be noticed that both investigated alloys are mainly comprised of α-Mg and Mg_6_Zn_3_Ca_2_ phases.

It was identified by Jiang et al. [62], Schäublin et al. [63], and Farahany et al. [64] that for Mg–Zn–Ca–Mn alloys, in the case of a calcium content higher than 0.5% wt%. three phases such as α-Mg, Mg_6_Zn_3_Ca_2_, and Mg_2_Ca can be observed. Also, when the Zn/Ca ratio becomes higher than 1.23, only α-Mg and Mg_6_Zn_3_Ca_2_ can be evidenced through XRD investigations even if the calcium content is higher than the limit mentioned above [65,66,67]. In the case of our samples, because the Zn/Ca ratio is higher than 1.23, the phase Mg_2_Ca cannot be identified in the spectra.

SEM and EDS investigations showed the presence of Mn homogenously distributed within the grains, similar to the description made by Cho et al. [68]. Usually, the alloy microstructure consists of (α-Mg + Mg_6_Zn_3_Ca_2_ + α-Mn) at a fabrication temperature interval between 230 °C and 360 °C, but the phase α-Mn cannot be evidenced on our samples due to its trace addition to the alloys. Kavyani et al. [60] manufactured by the stir casting method Mg–Zn–Ca–Mn alloys and analyzed the microstructure refinement, corrosion, and mechanical properties. They applied a plastic deformation procedure to improve the material’s properties. Also, Mg_2_Ca and α-Mn could not be identified in their study. The Mn content was about 0.75 wt%, and the Zn/Ca ratio was equal to 7.9. On the contrary, Bakhsheshi-Rad et al. [69] identified in Mg–2Ca–0.5Mn–2Zn (Zn/Ca ratio of 1.06 and a Ca content of 2.21 wt%) the presence of Mg_2_Ca concomitantly with α-Mg and Mg_6_Zn_3_Ca_2_. They could not detect in the XRD spectra the α-Mn peaks. Due to its trace character, it cannot be evidenced through diffraction or optical microscopy; only scanning electron microscopy can detect Mn presence.

**Figure 3:**
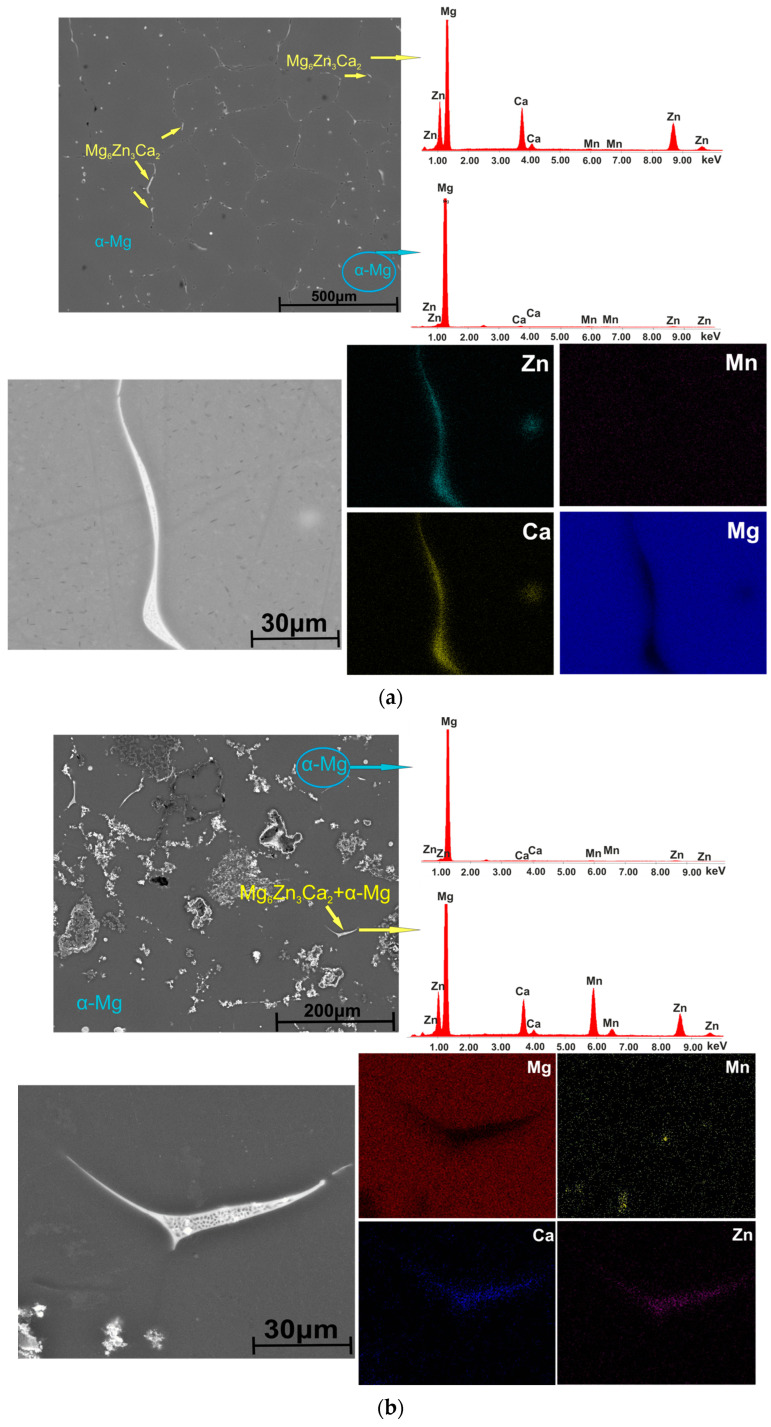
SEM images and corresponding EDS results for (**a**) ZMX100 and (**b**) ZMX410 alloy.

Scanning electron microscopy images coupled with EDS spectrometry on the experimental magnesium alloys after etching are presented in Figure 3.

The SEM images highlight the distribution and morphology of the secondary phases present in the investigated ZMX100 and ZMX410 alloys. One of these is found mainly at the grain boundary in a strip-like form. The chemical composition of these secondary phases was put into evidence through EDS analysis. In the case of ZMX100 alloy, the elements Mg, Zn, and Ca were identified at the grain boundary. In addition, the results show that Mn is uniformly distributed inside the grains as a dissolved element. No intermetallic compound of Mn is observed. In ZMX410 alloy at the grain boundary, a high concentration of Mn was highlighted in the form of granular compounds in addition to Mg, Zn, and Ca elements. Also, the granular inclusion of manganese could be observed in the α-Mg matrix. Based on these observations and taking into account the phases identified by XRD analysis, we can say that in the alloys’ structure next to the α-Mg phase, the formation of a eutectic can be observed at the grain boundary (α-Mg + Mg_6_Zn_3_Ca_2_) for ZMX100 alloy and (α-Mg + Mg_6_Zn_3_Ca_2_ + α-Mn) for ZMX410, respectively.

**Figure 4:**
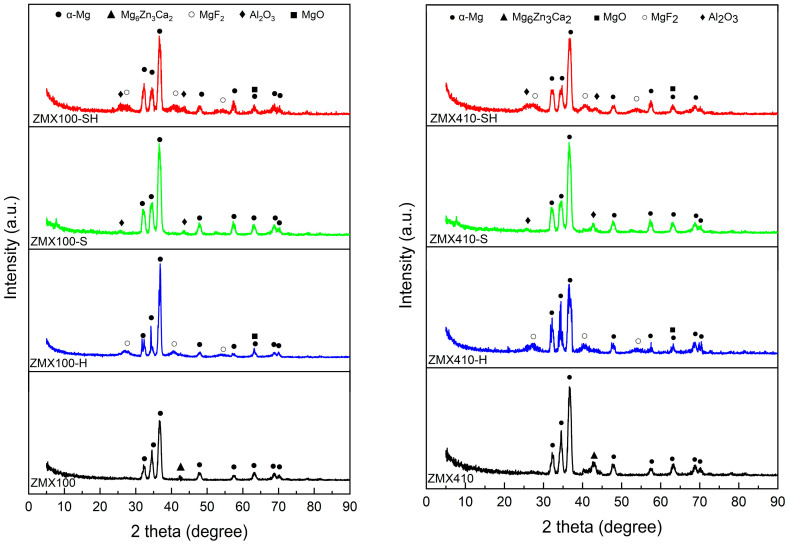
X-ray diffraction patterns of the untreated and treated ZMX100 and ZMX410 alloys.

In order to identify the specific structural phases for all untreated and treated experimental alloys (ZMX100 and ZMX410), XRD analysis was performed. The results are shown in Figure 4.

Along with the specific phases identified in ZMX100 and ZMX410 alloys’ XRD spectra (α-Mg + Mg_6_Zn_3_Ca_2_) for the ZMX100-H and ZMX410-H samples treated with HF, the XRD patterns highlight the presence of the magnesium fluoride conversion layer (MgF_2_). In contrast, the sandblasted alloy samples (ZMX100-S and ZMX410-S) indicate the presence of the Al_2_O_3_ phase, which shows the existence of some residual alumina particles on the alloy surface.

MgF_2_ is formed on the surface of magnesium alloys through the reaction of the magnesium with hydrofluoric acid according to Equation (2). The MgF_2_ layer reduces the corrosion rate, ensuring a gradual in vivo and in vitro degradation of magnesium-based alloys [39,70,71,72]. It was also reported that through the formation of this layer, the accumulation of hydrogen gas and the localized increase in pH could be controlled and facilitate cell adhesion and proliferation [71,72,73,74,75,76,77]. At the same time, during the formation of the MgF_2_ layer, an oxidation reaction also occurs according to Equation (3). The amount of magnesium hydroxide formed depends on the concentration of the hydrofluoric acid solution used [78].

(2) $${\mathrm{Mg}}_{\left(s\right)}+2{\mathrm{HF}}_{\left(\mathrm{aq}\right)}\to {\mathrm{MgF}}_{2\left(s\right)}+{\;H}_{2\left(g\right)}$$

(3) $${\mathrm{Mg}}_{\left(s\right)}+2H_{2}O_{\left(l\right)\;}\to \;\mathrm{Mg}{\left(\mathrm{OH}\right)}_{2\left(s\right)}+{\;H}_{2\left(g\right)}$$

Not being stable in the acid solution, the Mg(OH)_2_ layer undergoes the following transformations [71,78]:

(4) $$\mathrm{Mg}{\left(\mathrm{OH}\right)}_{2\left(s\right)}+2{\mathrm{HF}}_{\left(\mathrm{aq}\right)}\;\to {\;\mathrm{MgF}}_{2\left(s\right)}+2H_{2}O_{\left(l\right)}\;$$

(5) $$\mathrm{Mg}{\left(\mathrm{OH}\right)}_{2\left(s\right)}\;\to {\mathrm{MgO}}_{\left(s\right)}+H_{2}O_{\left(l\right)}$$

Magnesium oxide was also identified in the XRD spectra of the HF-treated alloy samples (ZMX100-H, ZMX100-SH, ZMX410-H, ZMX410-SH). Since the MgF_2_ layer formed on the ZMX100 and ZMX410 alloys surface is very thin in the XRD spectra, the alloy substrate was also detected (α-Mg and Mg_6_Zn_3_Ca_2_ phases).

**Figure 5:**
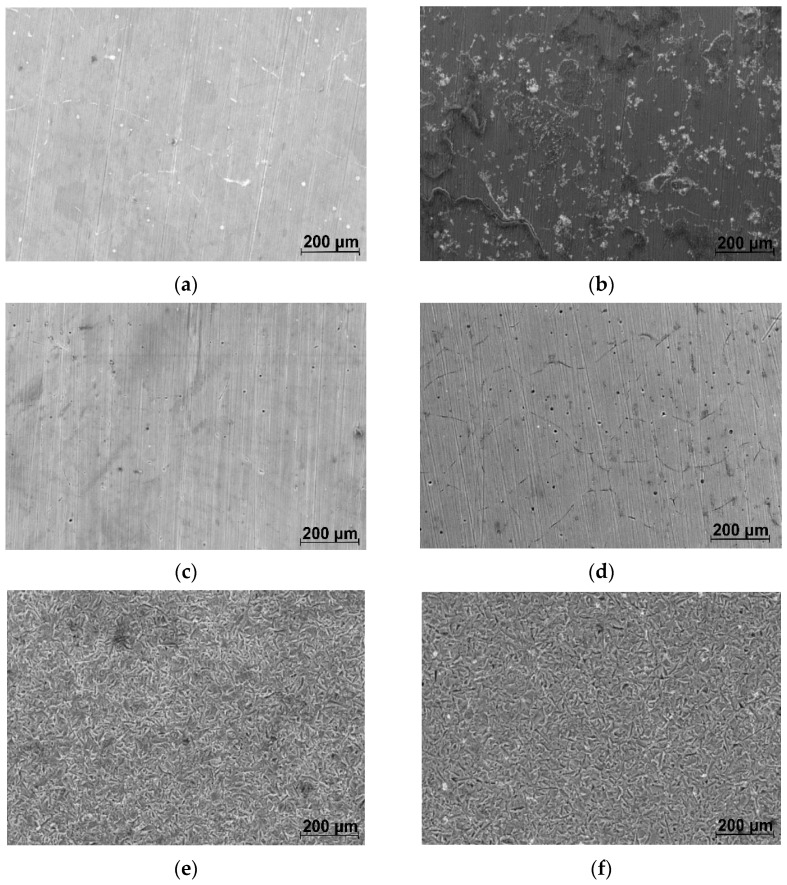

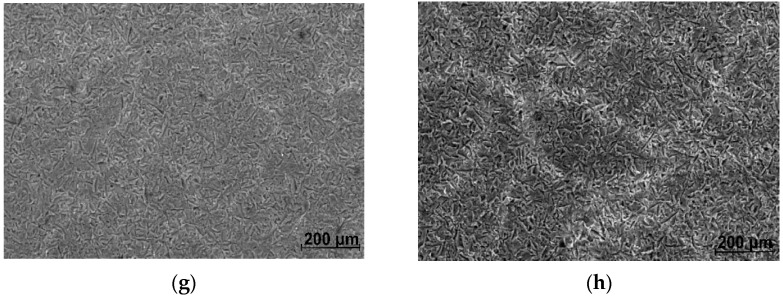
SEM images obtained for untreated alloys ((**a**) ZMX100, (**b**) ZMX410), fluoride treated alloys ((**c**) ZMX100-H, (**d**) ZMX410-H), sandblasted alloys ((**e**) ZMX100-S, (**f**) ZMX410-S) and combined treated alloys ((**g**) ZMX100-SH, (**h**) ZMX410-SH)—scalebars are 200 microns.

The morphology of the ZMX100 and ZMX410 alloy surfaces before and after applying the surface modification processes is shown in Figure 5.

In the case of HF-treated ZMX100 and ZMX410 alloys (Figure 5c,d), a compact film with irregularly distributed pores can be observed on their surface. The pores in the MgF_2_ coating layer are generated by the hydrogen release upon initial contact of the Mg-based alloys with the hydrofluoric acid solution. But the hydrogen release is not intensive, and the substrate is not affected by the presence of the pores due to the precipitation of MgF_2_ particles [39]. The surface of the sandblasted ZMX100 and ZMX410 alloys (Figure 5e,f) shows deep cavities on the entire surface, thus increasing the roughness. The presence of these cavities on the sandblasted alloy surfaces increases the contact area between the samples’ surfaces and the corrosion medium (ZMX100-S and ZMX410-S), and the identification by XRD analysis of the Al_2_O_3_ particles embedded on their surfaces could increase the degradation rates. Also, the sandblasting process could negatively affect the MgF_2_ layer formation and therefore decrease the corrosion resistance of ZMX100-SH and ZMX410-SH samples compared to the ZMX100 and ZMX410 alloys treated with HF.

**Figure 6:**
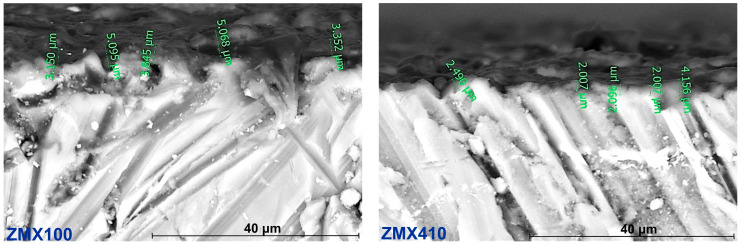
SEM images of the cross-section of the fluoride-treated ZMX100 and ZMX410 alloys.

Figure 6 shows the SEM images on the cross sections of fluoride-treated Mg-based alloy samples. These indicate that the fluoride coating adhered well to the substrate, with thickness layer values of about 3.5 μm for ZMX100 alloy and 2.0 μm for ZMX410 alloy, respectively. These values are in accordance with results published by other authors [70,71].

**Figure 7:**
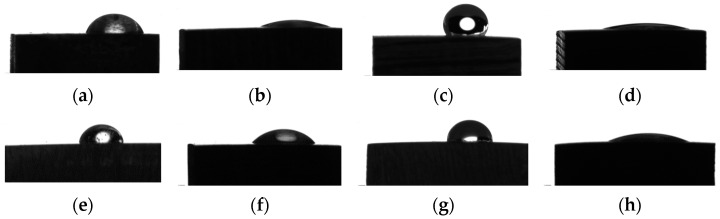
Droplet shapes and average values obtained for ZMX100 and ZMX410 alloys in the case of water. (**a**) ZMX100 sample (θ = 51.51 ± 0.76°), (**b**) ZMX100-H sample (θ = 14.94 ± 0.56°), (**c**) ZMX100-S sample (θ = 78.78 ± 0.50°), (**d**) ZMX100-SH sample (θ = 10.90 ± 0.59°), (**e**) ZMX410 sample (θ = 49.07 ± 0.53°), (**f**) ZMX410-H sample (θ = 14.45 ± 0.59°), (**g**) ZMX410-S sample (θ = 78.83 ± 0.71°), (**h**) ZMX410-SH sample (θ = 9.76 ± 0.25°).

**Figure 8:**
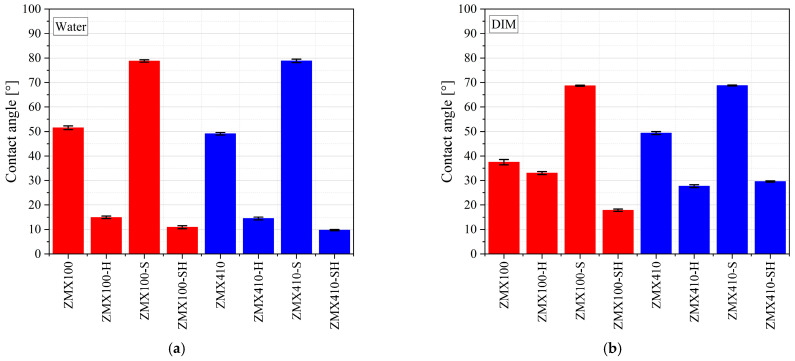

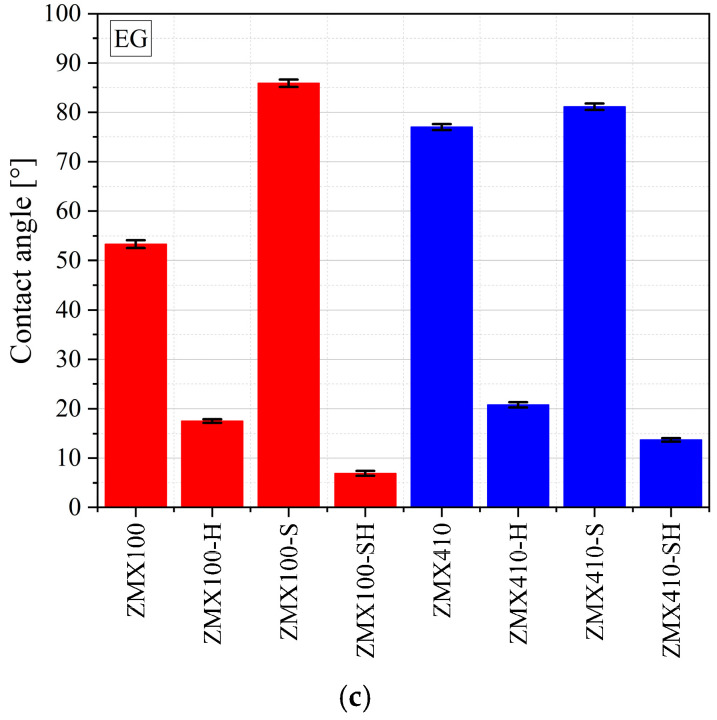
Comparison of contact angle representations for the two Mg-based alloys in the case of different test liquids: (**a**) Water, (**b**) Diiodomethane, (**c**) Ethylene glycol.

The wettability of the two investigated Mg-based alloys is important for a proper biological response after sample implantation. In the case of adequate hydrophilicity, cell adhesion and proliferation increase, and new bone formation occurs at the interface between the biological environment and material. A low contact angle value (θ < 90°) defines a hydrophilic surface favorable to molecules from biological fluid absorption. Figure 7 presents some examples of droplet shapes for the two investigated alloys in the case of water as a wetting agent for different surface treatments. It can be noticed that the sample treated with hydrofluoric acid (ZMX100-H and ZMX410-H) determines a decrease in the contact angle, while the sandblast treatment with Al_2_O_3_ particles (ZMX100-S and ZMX410-S) produces a more hydrophobic surface. The lowest contact angle value was observed for the combined surface treatment (sandblast followed by the HF treatment), which evidenced that this last procedure is adequate to produce hydrophilic surfaces favorable for biological integration. Figure 8 shows the graphs for treated and untreated ZMX100 and ZMX410 alloys obtained in the case of the three wetting agents. Water and ethylene glycol (EG) are considered polar liquids, and diiodomethane (DIM) is used as a nonpolar/dispersive liquid. Their surface energy components are known values, and they are reported in [50].

In the case of water as a wetting agent, the values of the contact angle increased for both investigated alloys after the sandblast surface treatment was applied (ZMX100-S and ZMX410-S, Figure 8). The contact angle decreased through sample immersion in HF, and the two investigated materials had a hydrophilic surface (ZMX100-H and ZMX410-H). It can be noticed that in the case of the combined surface treatment (sandblast followed by HF treatment), the sandblast effect is repealed by the HF treatment. For diiodomethane liquid, the HF surface treatment is more effective in the case of ZMX410 alloy, and the combined treatment leads to a more hydrophilic surface than in the case of ZMX100 material. The highest contact angle value was obtained for the ZMX100 sandblast surface measured in ethylene glycol. For EG as a wetting agent, the HF immersion of both samples (ZMX100-H and ZMX410-H) determines a decrease in the contact angle and an increase in the surface wettability.

**Figure 9:**
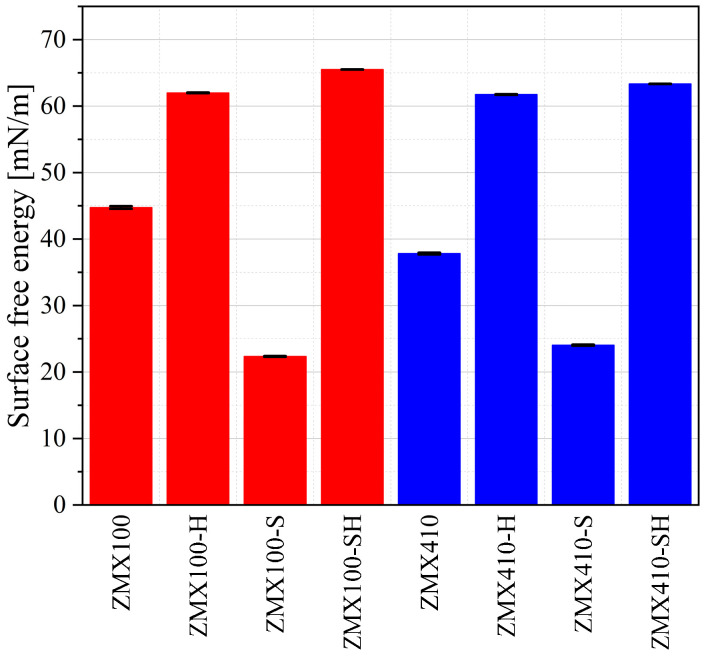
Surface Free Energy (SFE) results computed based on OWKR method for the two investigated Mg-based alloys.

In order to compute the alloy surface free energy (SFE), we applied the OWKR method. Based on the OWKR procedure described in [50] and known values of liquid surface free energy, we calculated the polar and dispersive components of the alloy SFE in the case of three samples/surface treatment. After that, an average value and standard deviation were considered the final results in each case. The total SFE was computed as the sum of the polar and dispersive interactions at the solid-liquid interface (Figure 9).

SFE can be correlated with wettability in a directly proportional manner. For higher values of SFE and lower values of contact angle, it can be concluded that there is an increased chance for biological factor adsorption and cell proliferation. In our case, the last surface treatment applied for both Mg-based alloys determines a high SFE, and it can be noticed that this treatment is suitable for implants, which follows to be involved in vivo analysis. Regarding the sandblast surface treatment, we obtained in both cases the lowest values of SFE, a fact that provides evidence that this type of treatment can be correlated with a stable state of energy and a low hydrophilicity property.

### 3.2. Corrosion Behavior

#### 3.2.1. Electrochemical Investigations

**Figure 10:**
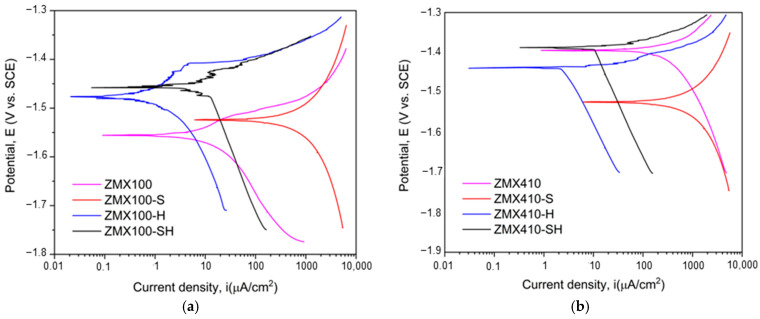
Tafel plots of both of the investigated Mg-based alloys: (**a**) ZMX100, (**b**) ZMX 410.

The Tafel curves for all the tested samples immersed in NaCl solution are presented in Figure 10.

The electrochemical parameters, such as corrosion potential (E_corr_), cathodic Tafel slope (β_c_), anodic Tafel slope (β_a_), and corrosion current density (i_corr_), were extracted from the dependencies shown in Figure 10 as presented in [2,79,80,81,82]. Based on the Stern-Geary relationship (Equation (6)) and in good accordance with ASTM G102-89 (2015) [83] (Equation (7)), the polarization resistance R_p_ and the corrosion rate (CR) were calculated. The open circuit potential (E_oc_) values of alloys were registered after 1 h of immersion before polarization resistance experiments. All the electrochemical parameters mentioned above are presented in Table 4.

(6) $$R_{p}=\frac{1}{2.3i_{\mathrm{corr}}}\frac{\beta_{a\;}\left|\beta_{c}\right|}{\beta_{a\;}+\left|\beta_{c}\right|}$$

(7) $$\mathrm{CR}=K_{i}\cdot \frac{i_{\mathrm{corr}}}{\rho }\cdot \mathrm{EW}$$

where K_i_ = 3.27 × 10^−1^ (C^−1^), EW represents the equivalent weight (g), and ρ is the material density (g/cm^3^).

The electrochemical measurements showed that when the NaCl solution is used as an electrolyte, all the samples exhibited negative values lower than −1 V for the open circuit and corrosion potentials. It is well known that a given material has good corrosion resistance when E_oc_ and E_corr_ have more electropositive values, small values of i_corr_, and a higher R_p_ value. According to this classification, it can be observed that the highest value of open circuit potential is obtained for ZMX100-H (−1.512 V) and ZMX410-H (−1.502 V), combined with the smallest corrosion current density of 5.81 μA/cm^2^ and 37.06 μA/cm^2^, respectively. Regarding the polarization resistance, the same samples were characterized by the highest values of 17.264 kΩcm^2^ (ZMX100-H) and 8.216 kΩcm^2^ (ZMX410-H). By analyzing further, the data presented in Table 4, it can be noticed that the sandblasted surface modification induced an increased value of the corrosion rate for both alloys of 13.204 mm/year (ZMX100-S) and 10.073 mm/year (ZMX410-S) because of the surface deep cavity apparition and high rugosity. The untreated samples presented the lowest value of E_oc_ (−1.577 V for ZMX100 and −1.506 V for ZMX410), but their corrosion behavior can be considered better in comparison with the sandblasted ones due to the fact that they exhibited lower values for i_corr_ (9.86 μA/cm^2^ for ZMX100 and 347.89 μA/cm^2^ for ZMX410, in comparison with 592.26 μA/cm^2^ and 469.55 μA/cm^2^, respectively). This observation is also sustained by the higher values of the polarization resistances determined in the case of untreated alloys.

Comparing the values presented in Table 4, due to the fact that ZMX410 alloy contains a higher weight percent of Zn (according to Table 2), it can be noticed that it corrodes faster. Also, Kavyani et al. [60] found that due to the galvanic couple that occurred between the Mg_6_Zn_3_Ca_2_ particles acting as a cathode and the Mg matrix exhibiting the behavior of an anode, the corrosion process and dissolution rate of the material were accelerated. These two phases were evidenced through XRD measurements for the investigated alloys, and an increased corrosion rate was obtained for ZMX410 compared with ZMX100 alloy [84].

In the Tafel curves (Figure 10), we can observe that the corrosion current shifted in a much less noble direction by increasing the Zn content. The cathodic polarisation curves are usually associated with the hydrogen evolution through the water reduction process since the anodic curves represent the Mg dissolution [69]. The polarisation resistance has a lower value (0.073 kΩcm^2^) and a higher corrosion rate (7.463 mm/year) for ZMX410 in comparison with ZMX100 (4.936 kΩcm^2^, 0.219 mm/year), showing evidence of poor corrosion behavior. Some literature studies stated that the Mg_2_Ca phase could improve the corrosion resistance of Mg–Zn–Ca–Mn alloys when the Zn/Ca ratio is below 1.25. In our case, this phase did not occur because the Zn/Ca ratio is equal to 2.08 for ZMX100 and 9.11 in the case of ZMX410 alloy, and supplementary, the Ca content is lower than 0.5%. In the absence of the phase mentioned above due to the galvanic coupling that appears in the material, the α-Mg corrodes faster since the Ca_2_Mg_6_Zn_3_ remains in the material structure and the α-Mg cannot further support it, which determines a decrease in the corrosion resistance when the Zn content increases.

It can be concluded that the surface modification obtained after hydrofluoric acid treatment leads to the highest corrosion resistance in the case of both tested alloys (i.e., the smallest values for i_corr_, the highest value of R_p_, and the lowest value of CR). For the HF treatment, the ZMX410-H alloy corrosion rate is equal to 0.795 mm/year in comparison with 0.129 mm/year for ZMX100-H. Supplementary, its corrosion current density has a higher value (37.06 μA/cm^2^, ZMX410-H) than that obtained in the case of the other alloy (5.81 μA/cm^2^, ZMX100-H), underlying the fact that an increased Zn content determines a decrease in the corrosion resistance. Regarding the combined surface modification, it can be concluded that the hydrofluoric acid treatment has a stronger effect than the Al_2_O_3_ sandblasting procedure, and an increase in the corrosion resistance is observed (i.e., regarding i_corr_: 94.81 μA/cm^2^ for ZMX410-SH in comparison with 469.55 μA/cm^2^ for ZMX410-S). The other parameters (R_P_: 0.619 kΩcm^2^ for ZMX410-SH and 0.057 kΩcm^2^ in the case of ZMX410-S; CR: 2.034 mm/year for ZMX410-SH and 10.073 mm/year in the case of ZMX410-S) sustain this observation. In the case of the ZMX100 alloy, the finding mentioned above is still valid, and we can conclude that for both alloys, the best surface modification is the HF treatment, which can also reduce the negative effect of sandblasting and decrease the corrosion rate.

#### 3.2.2. Immersion Test

**Figure 11:**
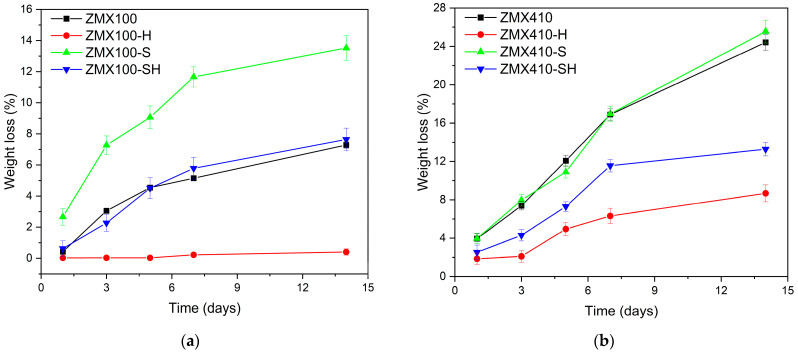
Weight loss of the untreated and treated ZMX100 (**a**) and ZMX410 (**b**) alloys after 14 days of immersion in NaCl solution.

The degradation behavior in NaCl solution for treated and untreated ZMX100 and ZMX410 alloys evaluated by weight loss is shown in Figure 11.

The degradation behavior of the ZMX100 and ZMX410 alloys using NaCl solution as a test medium reveals that HF treatment causes a decrease in weight loss for both investigated magnesium-based alloys. In the case of the ZMX100 alloy, it was observed that the MgF_2_ layer formed on the surface of the sample provides better protection compared to the ZMX410 alloy, with weight loss values after 14 days of immersion of 0.40% for ZMX100 and 8,67% for ZMX410, respectively. In the case of ZMX100, ZMX100-S, and ZMX100-SH samples, the weight loss is more accelerated in the first 7 days of immersion, after which the process is slowed down, probably due to the formation of corrosion products and environmental alkalinization. In the case of the ZMX410 alloys, this behavior was highlighted for the sample subjected to HF treatment (ZMX410-H) and combined treatment (sandblasting followed by HF treatment, ZMX410-SH). After the sandblasting process, the roughness of the substrate was modified, and the protective layer formed after HF treatment on sandblasted samples (ZMX100-SH and ZMX410-SH) appears to be less protective than in the case of samples subjected to only HF treatment (ZMX100-H and ZMX410-H).

The degradation behavior evaluated by the weight loss of the sandblasted samples (ZMX100-S and ZMX410-S) is significantly higher than that of those treated with HF (ZMX100-H and ZMX410-H). Due to the high concentration of Cl^−^ ions in the medium test, the layer of Mg(OH)_2_ that forms on the surface of the alloys is transformed into magnesium chloride (MgCl_2_), a compound soluble in the test medium (Equations (8)–(11)).

(8) $$\mathrm{Mg}\;\to {\;\mathrm{Mg}}^{2+}+2e^{-}$$

(9) $$2H_{2}O+2e^{-}\to {\;H}_{2}+2{\mathrm{HO}}^{-}$$

(10) $${\mathrm{Mg}}^{2+}+2{\mathrm{HO}}^{-}\;\to \mathrm{Mg}{\left(\mathrm{OH}\right)}_{2}$$

(11) $$\mathrm{Mg}{\left(\mathrm{OH}\right)}_{2}+2{\mathrm{Cl}}^{-}\;\to {\mathrm{MgCl}}_{2}+2{\mathrm{HO}}^{-}$$

The dissolution of the MgCl_2_ layer makes the alloy surface more active, so the biodegradation process intensifies. According to literature data [2,85,86], the corrosion process takes place until the solution pH reaches a value of 10 due to the accumulation of hydroxyl ions in the environment (Equation (11)). The high degradation behavior of sandblasted magnesium alloys is also due to their high roughness [87,88,89]. As in the other determinations, it can be observed that in the case of combined surface treatment (sandblasting followed by HF treatment), the negative effect of sandblasting is repealed by the HF treatment.

## 4. Conclusions

Following experimental research, a fluoride conversion coating was successfully obtained on both investigated Mg-based alloys, a layer composed of magnesium fluoride (MgF_2_) and magnesium oxide (MgO). The results of the electrochemical and immersion tests showed that the conversion layer generated on the surface of the alloys determines improved corrosion resistance. Also, high surface free energy values obtained for the alloys treated with hydrofluoric acid correlated with wettability lead to the conclusion that there is an increased chance for biological factor adsorption and cell proliferation.

For untreated alloys, the amount of the Mg_6_Zn_3_Ca_2_ phase plays an important role in the corrosion process. Thus, the higher the volume fraction of the Mg_6_Zn_3_Ca_2_ phase, the higher the corrosion rate. By increasing the percentage of Zn from 1.3 wt% in the ZMX100 alloy to 4.3 wt% in the case of the ZMX410 alloy, the volume of the Mg_6_Zn_3_Ca_2_ phase increased, resulting in a decrease in the corrosion resistance. Regarding the sandblasting treatment, this process increases the roughness of the alloys and strongly intensifies their corrosion process. Better results were obtained when the combined treatment was applied on both investigated alloys (sandblasting followed by hydrofluoric acid treatment), indicating that the hydrofluoric acid treatment has a stronger effect than the sandblasting procedure, thus generating an increase in corrosion resistance.

In conclusion, chemical conversion treatment with hydrofluoric acid is useful as a method of increasing corrosion resistance for the experimental Mg–Zn–Ca–Mn alloys.

## Figures and Tables

**Table 1:** Surface treatments that reduce the Mg-based alloy corrosion rate.

Mg-Based Alloys	Surface Modification	Remarks	Reference
Mg-3Sc-3Y	Self-passivation	The selective oxidation, made through the alloying process of Mg with Sc and Y, is a very effective way to control the Mg degradation rate	[33]
Mg-9Al-1Zn (AZ91)	Hydrothermal treatment	High corrosion resistance was put in evidence when AZ91 Mg material was immersed in phosphate-buffered saline (PBS) or Hank’s solutions	[34]
Pure Mg	Chemical passivation	The protective layer obtained in the case of 1 M NaOH was of the order of nanometers, and it has the following chemical composition MgO/Mg(OH)_2_	[24]
Phosphonic acid-derived self-assembled monolayers (SAMs) SAM Mg-3Al-1Zn (AZ31)	Self-assembled monolayers	Phosphonic acid-derived SAM AZ31 Mg-based material exhibits a higher corrosion resistance, and the chemical stability and adhesion of SAMs of alkyl phosphonic acid obtained after immersion technology was reported to be lower than that obtained through the vapor phase method	[31]
Mg–Ca	Fluoride conversion coatings	The MgF_2_ coating was prepared through Mg immersion in 200 g/L NaOH for 3 h, followed by conversion of Mg(OH)_2_ to MgF_2_ after immersion in 40% HF for 96 h	[39]
Mg-3Al-1Zn	Sol-gel coating	A TiO_2_ coating was prepared based on sol-gel coating. It was proven that TiO_2_ deposition increases the roughness of the AZ31 alloy to 0.133 nm and it decreases the contact angle value to about 20°, a fact that favored cell spreading and adhesion	[22]
Mg-9Al-1Zn (AZ91D)	Anodization	AZ91D material was anodized in a molybdate solution, and a biocompatible layer of about 70 μm was formed on the substrate surface when the applied voltage was 1 V. Ringer solution was used for corrosion tests, and the corrosion current density was reduced by 85% for the molybdenum-coated materials	[41]
Mg-4Y-3RE-0.5Zr (WE43)	Cathodic plasma electrolytic	Layers of Al_2_O_3_-Zr_2_O_2_ were deposited through this method on WE43Mg alloys based on a Al(NO_3_)_3_, Zr(NO_3_)_4_ and ethanol solution	[42]
Mg-9Al-1Zn (AZ91)	Physical vapor deposition	A HAp coating, with a thickness of 500 nm, was applied to AZ91 alloy, and a big improvement in corrosion resistance was obtained	[43]
Mg-4Y-3RE-0.5Zr (WE43)	Ion implantation	Zinc (Zn) and nitrogen (N) ions were implanted on a WE43 substrate. The corrosion resistance of these materials was substantially increased. The biocompatibility of the Mg-based alloys with a surface treatment made through ion implantation has been improved, a fact put in evidence by MC3T3-E1 cell high viability	[44]
Mg-3Al-1Zn (AZ31)	Surface mechanical attrition treatment	This surface treatment determines an increase in the micro-hardness and yield strength of the alloy	[46]
Mg-6Al-1Zn (AZ61)	Friction stir processing	An increased micro-hardness value and a reduced corrosion rate were found	[48]

**Table 2:** Chemical compositions of the investigated alloys (wt%).

Alloys	Zn	Mn	Ca	Mg	Zn/Ca (Atomic Ratio)
ZMX100	1.3	0.51	0.38	Bal.	2.08
ZMX410	4.3	0.62	0.30	Bal.	9.11

**Table 3:** Coding of experimental samples.

Samples	Treatment Applied
ZMX100	Original ZMX100 alloy
ZMX100-H	ZMX100 alloy treated with HF
ZMX100-S	Sandblasted ZMX100 alloy
ZMX100-SH	Sandblasted ZMX100 alloy treated with HF
ZMX410	Original ZMX410 alloy
ZMX410-H	ZMX410 alloy treated with HF
ZMX410-S	Sandblasted ZMX410 alloy
ZMX410-SH	Sandblasted ZMX410 alloy treated with HF

**Table 4:** Main electrochemical parameters.

Sample	E_oc_ (V)	E_corr_ (V)	i_corr_ (μA/cm^2^)	β_c_ (mV)	Βa (mV)	R_p_ (kΩcm^2^)	CR (mm/year)
ZMX100	−1.577	−1.556	9.86	158.54	381.95	4.936	0.219
ZMX100-H	−1.512	−1.476	5.81	339.20	723.19	17.264	0.129
ZMX100-S	−1.549	−1.523	592.26	114.07	90.61	0.037	13.204
ZMX100-SH	−1.552	−1.457	13.92	226.35	24.70	0.695	0.310
ZMX410	−1.506	−1.394	347.89	209.33	81.62	0.073	7.463
ZMX410-H	−1.502	−1.439	37.06	2222.00	1023.00	8.216	0.795
ZMX410-S	−1.504	−1.390	469.55	366.07	74.48	0.057	10.073
ZMX410-SH	−1.504	−1.388	94.81	1487.00	148.58	0.619	2.034

## Data Availability

Not applicable.

## References

[1] Antoniac I., Miculescu M., Mănescu V., Stere A., Quan P.H., Păltânea G., Robu A., Earar K. (2022). Magnesium-Based Alloys Used in Orthopedic Surgery. Materials.

[2] Quan P.H., Antoniac I., Miculescu F., Antoniac A., Manescu V., Robu A., Bița A.-I., Miculescu M., Saceleanu A., Bodog A.D. (2022). Fluoride Treatment and In Vitro Corrosion Behavior of Mg–Nd–Y–Zn–Zr Alloys Type. Materials.

[3] Robu A., Ciocoiu R., Antoniac A., Antoniac I., Raiciu A.D., Dura H., Forna N., Cristea M.B., Carstoc I.D. (2022). Bone Cements Used for Hip Prosthesis Fixation: The Influence of the Handling Procedures on Functional Properties Observed during In Vitro Study. Materials.

[4] Milea G.C., Necsulescu D.A., Ghiban B., Stere A., Robu A., Bujor C., Ene R., Forna N. (2022). Failure Analyses of a Non-Cemented Hip Prostheses Failed Due to the Stem Fracture. U.P.B. Sci. Bull. Ser. B.

[5] Lévesque J., Hermawan H., Dubé D., Mantovani D. (2008). Design of a Pseudo-Physiological Test Bench Specific to the Development of Biodegradable Metallic Biomaterials. Acta Biomater..

[6] (2022). Michael Jiang in This Paper, We Understand the Advantages and Disadvantages of Magnesium Alloys. https://news.metal.com.

[7] Zhang T., Wang W., Liu J., Wang L., Tang Y., Wang K. (2022). A Review on Magnesium Alloys for Biomedical Applications. Front. Bioeng. Biotechnol..

[8] Jung O., Porchetta D., Schroeder M.-L., Klein M., Wegner N., Walther F., Feyerabend F., Barbeck M., Kopp A. (2019). In Vivo Simulation of Magnesium Degradability Using a New Fluid Dynamic Bench Testing Approach. Int. J. Mol. Sci..

[9] Vlasie A., Trifu S., Lupuleac C., Kohn B., Cristea M. (2021). Restless Legs Syndrome: An Overview of Pathophysiology, Comorbidities and Therapeutic Approaches (Review). Exp. Ther. Med..

[10] Yin Yee Chin P., Cheok Q., Glowacz A., Caesarendra W. (2020). A Review of In-Vivo and In-Vitro Real-Time Corrosion Monitoring Systems of Biodegradable Metal Implants. Appl. Sci..

[11] Tizu M., Mărunțelu I., Cristea B.M., Nistor C., Ishkitiev N., Mihaylova Z., Tsikandelova R., Miteva M., Caruntu A., Sabliov C. (2022). PLGA Nanoparticles Uptake in Stem Cells from Human Exfoliated Deciduous Teeth and Oral Keratinocyte Stem Cells. J. Funct. Biomater..

[12] Rădulescu I., Drăgoi A., Trifu S., Cristea M. (2021). Neuroplasticity and Depression: Rewiring the Brain’s Networks through Pharmacological Therapy (Review). Exp. Ther. Med..

[13] Crișan R.-M., Băcilă C.I., Morar S. (2022). The Role of Psychological Autopsy in Investigating a Case of Atypical Suicide in Schizophrenia: A Case Report with a Brief Review of Literature. Egypt. J. Forensic Sci..

[14] Bohner M., Lemaitre J. (2009). Can Bioactivity Be Tested in Vitro with SBF Solution?. Biomaterials.

[15] Gonzalez J., Hou R.Q., Nidadavolu E.P.S., Willumeit-Römer R., Feyerabend F. (2018). Magnesium Degradation under Physiological Conditions—Best Practice. Bioact. Mater..

[16] Yang P., Ye S., Feng B., Liu J., Huang S., Liu G., Zhang W., Tang W., Zhu S., Zhang S. (2021). Microgalvanic Corrosion of Mg–Ca and Mg–Al–Ca Alloys in Nacl and Na2so4 Solutions. Materials.

[17] Liu W., Cao F., Xia Y., Chang L., Zhang J. (2014). Localized Corrosion of Magnesium Alloys in NaCl Solutions Explored by Scanning Electrochemical Microscopy in Feedback Mode. Electrochim. Acta.

[18] Hornig R., Mandel M., Krüger L., Bräunling S. (2022). Study of an Mg90Y7Zn3 Alloy (WZ73) in Sodium Chloride Solution—An Analysis by Correlated Polarization and Climate Chamber Testing. Eng. Rep..

[19] Esmaily M., Svensson J.E., Fajardo S., Birbilis N., Frankel G.S., Virtanen S., Arrabal R., Thomas S., Johansson L.G. (2017). Fundamentals and Advances in Magnesium Alloy Corrosion. Prog. Mater. Sci..

[20] Naddaf Dezfuli S., Huan Z., Mol J.M.C., Leeflang M.A., Chang J., Zhou J. (2014). Influence of HEPES Buffer on the Local PH and Formation of Surface Layer during in Vitro Degradation Tests of Magnesium in DMEM. Prog. Nat. Sci. Mater. Int..

[21] Zainal Abidin N.I., Rolfe B., Owen H., Malisano J., Martin D., Hofstetter J., Uggowitzer P.J., Atrens A. (2013). The in Vivo and in Vitro Corrosion of High-Purity Magnesium and Magnesium Alloys WZ21 and AZ91. Corros. Sci..

[22] Xin Y., Hu T., Chu P.K. (2010). Influence of Test Solutions on In Vitro Studies of Biomedical Magnesium Alloys. J. Electrochem. Soc..

[23] Ellison G., Straumfjord J.V., Hummel J.P. (1958). Buffer Capacities of Human Blood and Plasma. Clin. Chem..

[24] Lorenz C., Brunner J.G., Kollmannsberger P., Jaafar L., Fabry B., Virtanen S. (2009). Effect of Surface Pre-Treatments on Biocompatibility of Magnesium. Acta Biomater..

[25] Galvin E., Jaiswal S., Lally C., MacDonald B., Duffy B. (2017). In Vitro Corrosion and Biological Assessment of Bioabsorbable WE43 Mg Alloy Specimens. J. Manuf. Mater. Process..

[26] Miculescu M., Ion O.A. (2022). Regulation and Certification of (Bio)Medical Engineers: A Case Study on Romania. Int. J. Environ. Res. Public Health.

[27] Wang J., Giridharan V., Shanov V., Xu Z., Collins B., White L., Jang Y., Sankar J., Huang N., Yun Y. (2014). Flow-Induced Corrosion Behavior of Absorbable Magnesium-Based Stents. Acta Biomater..

[28] Marco I., Feyerabend F., Willumeit-Römer R., Van der Biest O. (2016). Degradation Testing of Mg Alloys in Dulbecco’s Modified Eagle Medium: Influence of Medium Sterilization. Mater. Sci. Eng. C.

[29] Bowen P.K., Drelich J., Goldman J. (2013). A New in Vitro–in Vivo Correlation for Bioabsorbable Magnesium Stents from Mechanical Behavior. Mater. Sci. Eng. C.

[30] Amaravathy P., Rose C., Sathiyanarayanan S., Rajendran N. (2012). Evaluation of in Vitro Bioactivity and MG63 Oesteoblast Cell Response for TiO_2_ Coated Magnesium Alloys. J. Solgel Sci. Technol..

[31] Ishizaki T., Teshima K., Masuda Y., Sakamoto M. (2011). Liquid Phase Formation of Alkyl- and Perfluoro-Phosphonic Acid Derived Monolayers on Magnesium Alloy AZ31 and Their Chemical Properties. J. Colloid Interface Sci..

[32] Hermawan H. (2012). Biodegradable Metals.

[33] Brar H.S., Ball J.P., Berglund I.S., Allen J.B., Manuel M.V. (2013). A Study of a Biodegradable Mg–3Sc–3Y Alloy and the Effect of Self-Passivation on the in Vitro Degradation. Acta Biomater..

[34] Zhu Y., Wu G., Zhang Y.-H., Zhao Q. (2011). Growth and Characterization of Mg(OH)_2_ Film on Magnesium Alloy AZ31. Appl. Surf. Sci..

[35] Latham J.-A., Howlett P.C., MacFarlane D.R., Forsyth M. (2011). Electrochemical Reactivity of Trihexyl(Tetradecyl)Phosphonium Bis(2,4,4-Trimethylpentyl)Phosphinate Ionic Liquid on Glassy Carbon and AZ31 Magnesium Alloy. Electrochim. Acta.

[36] Cui X., Yang Y., Liu E., Jin G., Zhong J., Li Q. (2011). Corrosion Behaviors in Physiological Solution of Cerium Conversion Coatings on AZ31 Magnesium Alloy. Appl. Surf. Sci..

[37] Hu L., Meng Q., Chen S., Wang H. (2012). Effect of Zn Content on the Chemical Conversion Treatments of AZ91D Magnesium Alloy. Appl. Surf. Sci..

[38] Drábiková J., Fintová S., Tkacz J., Doležal P., Wasserbauer J. (2017). Unconventional Fluoride Conversion Coating Preparation and Characterization. Anti-Corros. Methods Mater..

[39] Drynda A., Seibt J., Hassel T., Bach F.W., Peuster M. (2013). Biocompatibility of Fluoride-Coated Magnesium-Calcium Alloys with Optimized Degradation Kinetics in a Subcutaneous Mouse Model. J. Biomed. Mater. Res. A.

[40] Agarwal S., Curtin J., Duffy B., Jaiswal S. (2016). Biodegradable Magnesium Alloys for Orthopaedic Applications: A Review on Corrosion, Biocompatibility and Surface Modifications. Mater. Sci. Eng. C.

[41] Forero López A.D., Lehr I.L., Saidman S.B. (2017). Anodisation of AZ91D Magnesium Alloy in Molybdate Solution for Corrosion Protection. J. Alloys Compd..

[42] Liu P., Pan X., Yang W., Cai K., Chen Y. (2012). Al_2_O_3_–ZrO_2_ Ceramic Coatings Fabricated on WE43 Magnesium Alloy by Cathodic Plasma Electrolytic Deposition. Mater. Lett..

[43] Melnikov E.S., Surmeneva M.A., Tyurin A.I., Pirozhkova T.S., Shuvarin I.A., Prymak O., Epple M., Surmenev R.A. (2017). Improvement of the Mechanical Properties of AZ91D Magnesium Alloys by Deposition of Thin Hydroxyapatite Film. Nano Hybrids Compos..

[44] Wu G., Gong L., Feng K., Wu S., Zhao Y., Chu P.K. (2011). Rapid Degradation of Biomedical Magnesium Induced by Zinc Ion Implantation. Mater. Lett..

[45] Wu G. (2007). Fabrication of Al and Al/Ti Coatings on Magnesium Alloy by Sputtering. Mater. Lett..

[46] Zhang Z., Li Y., Peng J., Guo P., Huang J., Yang P., Wang S., Chen C., Zhou W., Wu Y. (2019). Combining Surface Mechanical Attrition Treatment with Friction Stir Processing to Optimize the Mechanical Properties of a Magnesium Alloy. Mater. Sci. Eng. A.

[47] Arun Kumar R., Ramesh S., Kedarvignesh E.S., Aravind Arulchelvam M.S., Anjunath S. (2019). Review of Friction Stir Processing of Magnesium Alloys. Mater. Today Proc..

[48] Sithole L.M., Madushele N. (2019). Surface Treatment of Magnesium AZ61 Alloy with Stainless Steel Powder by Friction Stir Processing. Procedia Manuf..

[49] Rahim S.A., Joseph M.A., Sampath Kumar T.S. (2022). Recent Progress in Surface Modification of Mg Alloys for Biodegradable Orthopedic Applications. Front. Mater..

[50] Annamalai M., Gopinadhan K., Han S.A., Saha S., Park H.J., Cho E.B., Kumar B., Patra A., Kim S.-W., Venkatesan T. (2016). Surface Energy and Wettability of van Der Waals Structures. Nanoscale.

[51] Huan Z.G., Leeflang M.A., Zhou J., Fratila-Apachitei L.E., Duszczyk J. (2010). In Vitro Degradation Behavior and Cytocompatibility of Mg–Zn–Zr Alloys. J. Mater. Sci. Mater. Med..

[52] Zhang B., Hou Y., Wang X., Wang Y., Geng L. (2011). Mechanical Properties, Degradation Performance and Cytotoxicity of Mg–Zn–Ca Biomedical Alloys with Different Compositions. Mater. Sci. Eng. C.

[53] Rosalbino F., De Negri S., Saccone A., Angelini E., Delfino S. (2010). Bio-Corrosion Characterization of Mg–Zn–X (X = Ca, Mn, Si) Alloys for Biomedical Applications. J. Mater. Sci. Mater. Med..

[54] Xu Z., Smith C., Chen S., Sankar J. (2011). Development and Microstructural Characterizations of Mg–Zn–Ca Alloys for Biomedical Applications. Mater. Sci. Eng. B.

[55] Zhao X., Shi L., Xu J. (2013). Biodegradable Mg–Zn–Y Alloys with Long-Period Stacking Ordered Structure: Optimization for Mechanical Properties. J. Mech. Behav. Biomed. Mater..

[56] Smith C.E., Xu Z., Waterman J., Sankar J. (2013). Cytocompatibility Assessment of MgZnCa Alloys. Emerg. Mater. Res..

[57] Mohan A.G., Ciurea A.V., Antoniac I., Manescu (Paltanea) V., Bodog A., Maghiar O., Marcut L., Ghiurau A., Bodog F. (2022). Cranioplasty after Two Giant Intraosseous Angiolipomas of the Cranium: Case Report and Literature Review. Healthcare.

[58] Bakhsheshi-Rad H.R., Idris M.H., Abdul-Kadir M.R., Ourdjini A., Medraj M., Daroonparvar M., Hamzah E. (2014). Mechanical and Bio-Corrosion Properties of Quaternary Mg–Ca–Mn–Zn Alloys Compared with Binary Mg–Ca Alloys. Mater. Des..

[59] Nakata T., Xu C., Ito Y., Kamado S. (2022). Role of Homogenization on Tensile Properties and Microstructures in a Dilute Mg–Zn–Ca–Mn Alloy Sheet. Mater. Sci. Eng. A.

[60] Kavyani M., Ebrahimi G.R., Ezatpour H.R., Jahazi M. (2022). Microstructure Refinement, Mechanical and Biocorrosion Properties of Mg–Zn–Ca–Mn Alloy Improved by a New Severe Plastic Deformation Process. J. Magnes. Alloys.

[61] Bazhenov V.E., Li A.V., Komissarov A.A., Koltygin A.V., Tavolzhanskii S.A., Bautin V.A., Voropaeva O.O., Mukhametshina A.M., Tokar A.A. (2021). Microstructure and Mechanical and Corrosion Properties of Hot-Extruded Mg–Zn–Ca–(Mn) Biodegradable Alloys. J. Magnes. Alloys.

[62] Jiang M.G., Xu C., Nakata T., Yan H., Chen R.S., Kamado S. (2016). Development of Dilute Mg–Zn–Ca–Mn Alloy with High Performance via Extrusion. J. Alloys Compd..

[63] Schäublin R.E., Becker M., Cihova M., Gerstl S.S.A., Deiana D., Hébert C., Pogatscher S., Uggowitzer P.J., Löffler J.F. (2022). Precipitation in Lean Mg–Zn–Ca Alloys. Acta Mater..

[64] Farahany S., Bakhsheshi-Rad H.R., Idris M.H., Abdul Kadir M.R., Lotfabadi A.F., Ourdjini A. (2012). In-Situ Thermal Analysis and Macroscopical Characterization of Mg–XCa and Mg–0.5Ca–XZn Alloy Systems. Thermochim. Acta.

[65] Du Y.Z., Zheng M.Y., Qiao X.G., Wu K., Liu X.D., Wang G.J., Lv X.Y. (2013). Microstructure and Mechanical Properties of Mg–Zn–Ca–Ce Alloy Processed by Semi-Continuous Casting. Mater. Sci. Eng. A.

[66] Du Y.Z., Zheng M.Y., Xu C., Qiao X.G., Wu K., Liu X.D., Wang G.J., Lv X.Y. (2013). Microstructures and Mechanical Properties of As-Cast and as-Extruded Mg-4.50Zn-1.13Ca (Wt%) Alloys. Mater. Sci. Eng. A.

[67] Levi G., Avraham S., Zilberov A., Bamberger M. (2006). Solidification, Solution Treatment and Age Hardening of a Mg–1.6wt.% Ca–3.2wt.% Zn Alloy. Acta Mater..

[68] Cho D.H., Nam J.H., Lee B.W., Cho K.M., Park I.M. (2016). Effect of Mn Addition on Grain Refinement of Biodegradable Mg 4Zn 0.5Ca Alloy. J. Alloys Compd..

[69] Bakhsheshi-Rad H.R., Hamzah E., Daroonparvar M., Kasiri-Asgarani M., Medraj M. (2014). Synthesis and Biodegradation Evaluation of Nano-Si and Nano-Si/TiO_2_ Coatings on Biodegradable Mg–Ca Alloy in Simulated Body Fluid. Ceram. Int..

[70] Yan T., Tan L., Zhang B., Yang K. (2014). Fluoride Conversion Coating on Biodegradable AZ31B Magnesium Alloy. J. Mater. Sci. Technol..

[71] Yan T., Tan L., Xiong D., Liu X., Zhang B., Yang K. (2010). Fluoride Treatment and in Vitro Corrosion Behavior of an AZ31B Magnesium Alloy. Mater. Sci. Eng. C.

[72] da Conceicao T.F., Scharnagl N., Blawert C., Dietzel W., Kainer K.U. (2010). Surface Modification of Magnesium Alloy AZ31 by Hydrofluoric Acid Treatment and Its Effect on the Corrosion Behaviour. Thin Solid Film.

[73] Chiu K.Y., Wong M.H., Cheng F.T., Man H.C. (2007). Characterization and Corrosion Studies of Fluoride Conversion Coating on Degradable Mg Implants. Surf. Coat. Technol..

[74] Pereda M.D., Alonso C., Burgos-Asperilla L., Del Valle J.A., Ruano O.A., Perez P., Fernández Lorenzo De Mele M.A. (2010). Corrosion Inhibition of Powder Metallurgy Mg by Fluoride Treatments. Acta Biomater..

[75] Thomann M., Krause C., Angrisani N., Bormann D., Hassel T., Windhagen H., Meyer-Lindenberg A. (2010). Influence of a Magnesium-Fluoride Coating of Magnesium-Based Implants (MgCa0.8) on Degradation in a Rabbit Model. J. Biomed. Mater. Res. A.

[76] Carboneras M., García-Alonso M.C., Escudero M.L. (2011). Biodegradation Kinetics of Modified Magnesium-Based Materials in Cell Culture Medium. Corros. Sci..

[77] Pereda M.D., Alonso C., Gamero M., Del Valle J.A., Fernández Lorenzo De Mele M. (2011). Comparative Study of Fluoride Conversion Coatings Formed on Biodegradable Powder Metallurgy Mg: The Effect of Chlorides at Physiological Level. Mater. Sci. Eng. C.

[78] Pourbaix M.J.N., De Zoubov N., Van Muylder J. (1963). Atlas d’Équilibres Électrochimiques.

[79] Bița A.-I., Antoniac I., Miculescu M., Stan G.E., Leonat L., Antoniac A., Constantin B., Forna N. (2022). Electrochemical and In Vitro Biological Evaluation of Bio-Active Coatings Deposited by Magnetron Sputtering onto Biocompatible Mg-0.8Ca Alloy. Materials.

[80] Antoniac I., Miculescu F., Cotrut C., Ficai A., Rau J.V., Grosu E., Antoniac A., Tecu C., Cristescu I. (2020). Controlling the Degradation Rate of Biodegradable Mg–Zn–Mn Alloys for Orthopedic Applications by Electrophoretic Deposition of Hydroxyapatite Coating. Materials.

[81] Istrate B., Rau J.V., Munteanu C., Antoniac I.V., Saceleanu V. (2020). Properties and in Vitro Assessment of ZrO_2_-Based Coatings Obtained by Atmospheric Plasma Jet Spraying on Biodegradable Mg–Ca and Mg–Ca–Zr Alloys. Ceram. Int..

[82] Rau J.V., Antoniac I., Filipescu M., Cotrut C., Fosca M., Nistor L.C., Birjega R., Dinescu M. (2018). Hydroxyapatite Coatings on Mg–Ca Alloy Prepared by Pulsed Laser Deposition: Properties and Corrosion Resistance in Simulated Body Fluid. Ceram. Int..

[83] (2006). Standard Practice for Calculation of Corrosion Rates and Related Information from Electrochemical Measurements—Annual Book of ASTM Standards.

[84] Cho D.H., Avey T., Nam K.H., Dean D., Luo A.A. (2022). In Vitro and in Vivo Assessment of Squeeze-Cast Mg–Zn–Ca–Mn Alloys for Biomedical Applications. Acta Biomater..

[85] Zhang S., Zhang X., Zhao C., Li J., Song Y., Xie C., Tao H., Zhang Y., He Y., Jiang Y. (2010). Research on an Mg–Zn Alloy as a Degradable Biomaterial. Acta Biomater..

[86] Zhang S., Li J., Song Y., Zhao C., Zhang X., Xie C., Zhang Y., Tao H., He Y., Jiang Y. (2009). In Vitro Degradation, Hemolysis and MC3T3-E1 Cell Adhesion of Biodegradable Mg–Zn Alloy. Mater. Sci. Eng. C.

[87] Nguyen T.L., Blanquet A., Staiger M.P., Dias G.J., Woodfield T.B.F. (2012). On the Role of Surface Roughness in the Corrosion of Pure Magnesium in Vitro. J. Biomed. Mater. Res. B Appl. Biomater..

[88] Walter R., Kannan M.B., He Y., Sandham A. (2013). Effect of Surface Roughness on the in Vitro Degradation Behaviour of a Biodegradable Magnesium-Based Alloy. Appl. Surf. Sci..

[89] Gawlik M., Wiese B., Desharnais V., Ebel T., Willumeit-Römer R. (2018). The Effect of Surface Treatments on the Degradation of Biomedical Mg Alloys—A Review Paper. Materials.

